# A Comprehensive Review on HIV-Associated Dermatologic Manifestations: From Epidemiology to Clinical Management

**DOI:** 10.1155/2023/6203193

**Published:** 2023-07-18

**Authors:** Zeinab Mohseni Afshar, Azadeh Goodarzi, Seyed Naser Emadi, Ronak Miladi, Safoura Shakoei, Alireza Janbakhsh, Zeinab Aryanian, Parvaneh Hatami

**Affiliations:** ^1^Clinical Research Development Center, Imam Reza Hospital, Kermanshah University of Medical Sciences, Kermanshah, Iran; ^2^Department of Dermatology, Rasool Akram Medical Complex Clinical Research Development Center, School of Medicine, Iran University of Medical Sciences, Tehran, Iran; ^3^Skin and Stem Cell Research Center, Tehran University of Medical Sciences, Tehran, Iran; ^4^Autoimmune Bullous Diseases Research Center, Razi Hospital, Tehran University of Medical Sciences, Tehran 1199663911, Iran; ^5^Department of Dermatology, School of Medicine, Razi Hospital, Tehran University of Medical Sciences, Tehran 1199663911, Iran; ^6^Dermatology Department of Imam Khomeini Complex Hospital, Tehran University of Medical Sciences, Tehran 1199663911, Iran; ^7^Department of Dermatology, Babol University of Medical Sciences, Babol, Iran

## Abstract

Dermatological disorders are among the most prevalent manifestations of HIV infection/acquired immunodeficiency syndrome (AIDS). In this review, we aimed to characterize the various dermatologic presentations among HIV-infected patients with a detailed categorization of the mucocutaneous signs and symptoms, their etiopathogenic factors, and clinical management. In fact, cutaneous manifestations of HIV are quite various, ranging from AIDS-specific skin eruptions (xerosis, pruritic papular eruptions, eosinophilic folliculitis, and acne), opportunistic infections (herpes simplex, molluscum contagiosum, cutaneous leishmaniasis, bacillary angiomatosis, disseminated histoplasmosis, disseminated cryptococcosis, and zoster) to AIDS-related malignancies (Kaposi's sarcoma, lymphoma, and nonmelanoma skin cancers) and antiretroviral therapy (ART)-associated drug eruptions. We tried to classify HIV-related cutaneous presentations which can help clinicians for a better understanding of the various specific and nonspecific features of AIDS-associated cutaneous manifestations and management of the condition.

## 1. Introduction

Dermatological disorders are among the most prevalent manifestations of HIV infection/acquired immunodeficiency syndrome (AIDS).

**Figure 1:**
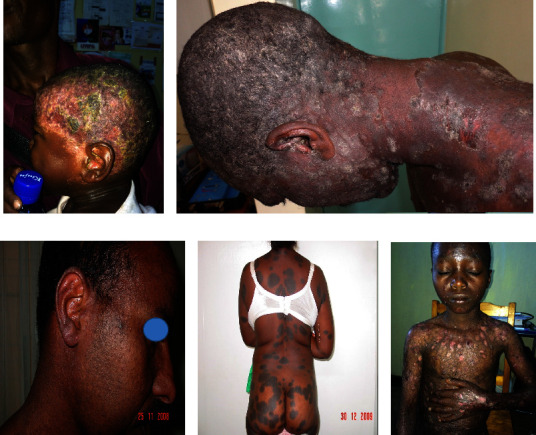
Some dermatoses in HIV-infected patients. Histiocytosis (a), pemphigus vulgaris (b), Darier's disease (c), generalized morphea (d), and epidermodysplasia verruciformis (*EV*) (e). Photos were taken by Dr. Emadi.

**Figure 2:**
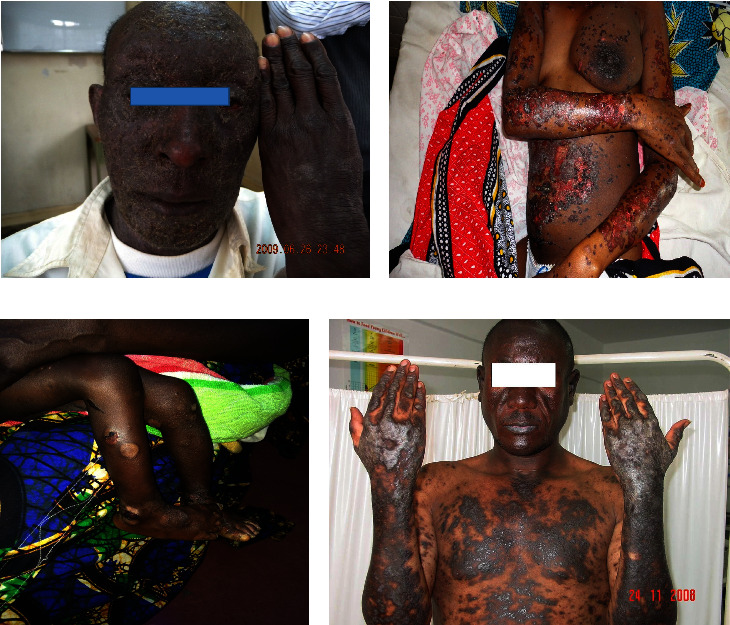
Some dermatoses in HIV-infected patients. Severe chronic actinic dermatitis (a), severe eczema (b), epidermolysis bullosa (c), and hypertrophic lichen planus (d). Photos were taken by Dr. Emadi.

**Figure 3:**
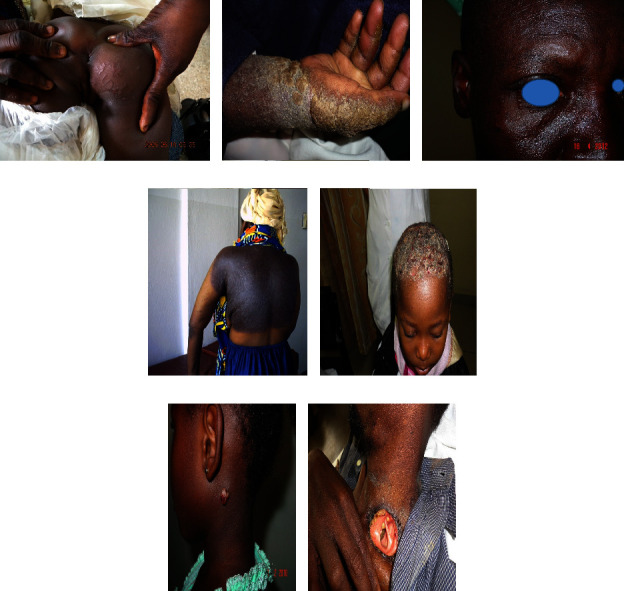
Some infectious disorders in HIV-positive patients: cutaneous larva migrans (a), crusted scabies (b), cryptococcal infection (c), dermatophytosis (d, e), bacillary angiomatosis (f), and scrofuloderma (g). Photos were taken by Dr. Emadi.

**Figure 4:**
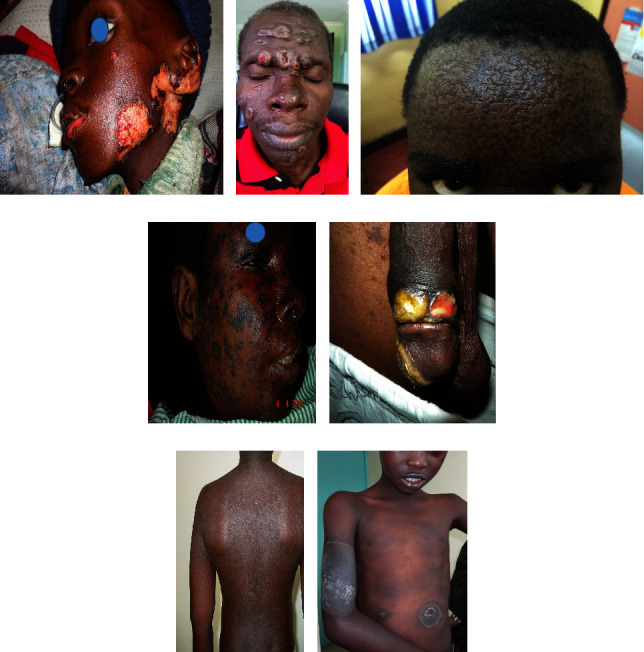
Some infectious disorders in HIV-positive patients: herpes simplex infection (a), giant molluscum contagiosum (b), plane wart (c), chicken pox (d), chancroid (e), the second phase of syphilis (f), and dermatophytosis (g). Photos were taken by Dr. Emadi.

**Figure 5:**
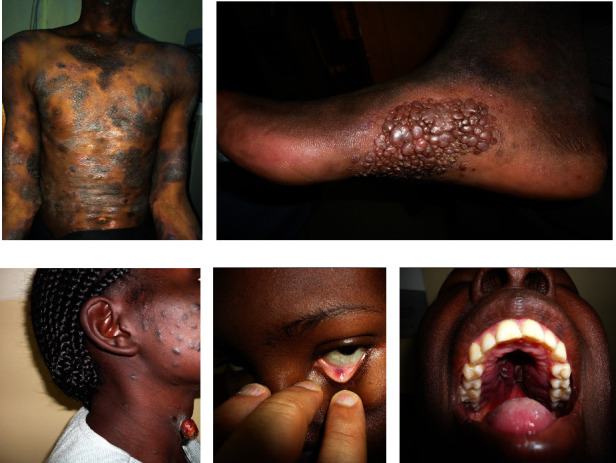
Some malignancies in HIV-infected patients: mycosis fungoides (a) and Kaposi sarcoma (b–e). Photos were taken by Dr. Emadi.

**Figure 6:**
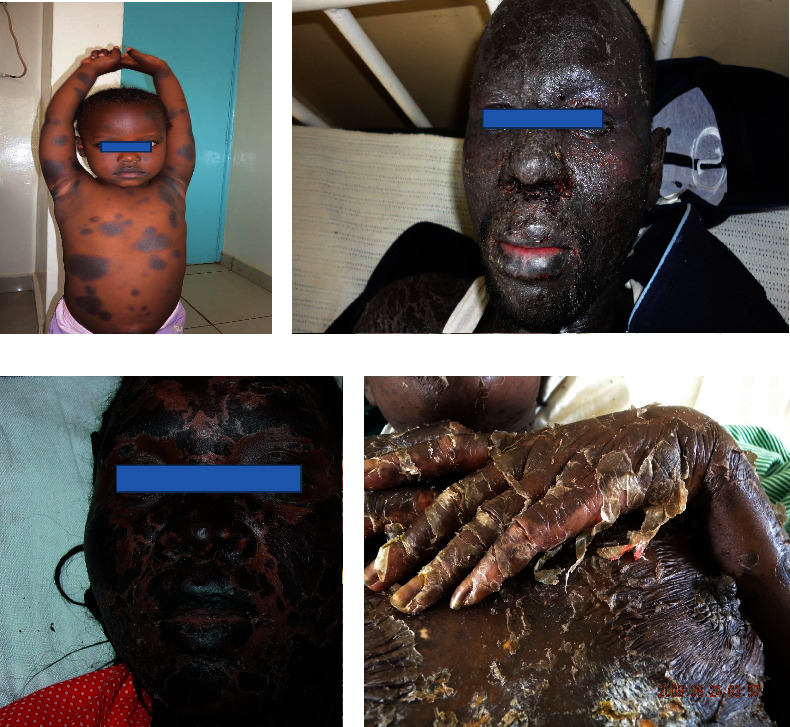
Some drug eruptions in HIV-infected patients: fixed drug eruption (FDE) due to cotrimoxazole (a), Stevens-Johnson syndrome (SJS) due to nevirapine (b), toxic epidermal necrolysis (TEN) due to cotrimoxazole (c), and TEN due to efavirenz (d). Photos were taken by Dr. Emadi.

Cutaneous manifestations of HIV are quite various, ranging from AIDS-specific skin eruptions (xerosis, pruritic papular eruptions, eosinophilic folliculitis, and acne), opportunistic infections (herpes simplex, molluscum contagiosum, cutaneous leishmaniasis, bacillary angiomatosis, disseminated histoplasmosis, disseminated cryptococcosis, and zoster) to AIDS-related malignancies (Kaposi's sarcoma, lymphoma, and nonmelanoma skin cancers) and antiretroviral therapy (ART)-associated drug eruptions. In Figures 1–6, some of these manifestations are presented.

The evolution of ART has also led to the introduction of the immune reconstitution inflammatory syndrome (IRIS) which is usually manifested as skin involvement. Moreover, other sexually transmitted diseases (STDs) (scabies, condyloma acuminatum, and syphilis) acquired with the same mode of HIV transmission, intravenous drug user (IVDU)-associated complications (abscess and infective endocarditis), and poor lifestyle-associated events (arthropod bites, lice, and scabies) can lead to cutaneous manifestations. On the other hand, usual skin infections in immunocompetent individuals (tinea, verruca vulgaris, bacterial cellulitis, and staphylococcal scalded skin syndrome) can also occur in HIV-infected patients with equal or higher frequency. In addition, some dermatoses (seborrheic dermatitis, psoriasis, urticaria, and vitiligo) seem to occur more prevalently in the settings of HIV infection [1]. Last but not the least, HIV-related vasculitis can also cause dermatologic involvement. Most of the cutaneous disorders in the context of HIV/AIDS are managed easily in outpatient settings; however, some of them cause significant morbidity and mortality [2, 3]. Here, we have discussed every aspect of association of HIV infection and dermatology in detail.

## 2. Methods

We searched on PubMed, Google Scholar, and Scopus in this regard, and all of the relevant papers published until January, 2023, were included if we could access their full-texts. Moreover, we included photos of some cases visited by one of the authors of this article (N. E) at South Africa.

## 3. Results and Discussion

We found various mucocutaneous manifestations associated with HIV and categorized them regarding their specificity and ethiopathogenesis.

### 3.1. HIV-Specific Dermatoses

Papular pruritic eruption (PPE), which is the most prevalent dermatologic manifestation in HIV-infected patients, is characterized by chronic symmetric papular eruptions on the limbs, trunk, and sometimes face. The exact etiology of PPE is not yet identified, but it has been demonstrated that lower CD4 cell counts are correlated with more intense pruritus and increased rash severity [4].

Xeoderma or xerosis occurs due to impairment in barrier function of stratum corneum, which occurs as a result of lipid content decrease within the viable epidermis and dermis and excessive concentration of carotenoids, especially lycopene, within the epidermis. This dermatologic condition is the main cause of premature skin ageing in HIV patients and one of the reasons of pruritus in these patients [5].

Eosinophilic folliculitis has been reported most commonly in HIV patients, especially those with lower CD4 counts. This eruption is thought to be a hypersensitivity response to some antigens in the pilosebaceous glands. Furthermore, autoimmunity, Pityrosporum yeast, and some bacteria may also play a role in its pathogenesis [6].

Prurigo nodularis (PN), also known as picker's nodule, has been predominantly reported in HIV patients [7].

Papular mucinosis (PM), also known as localized lichen myxedematosus (LM), is a kind of cutaneous mucinosis, whose correlation with HIV infection has been documented [8].

Any type of acne, including vulgaris, rosacea, and conglobata, can occur in the settings of IRIS after starting the patient on ART [9]. Moreover, acne conglobata, pityriasis rubra pilaris, and elongated follicular spines can appear simultaneously as components of HIV-associated follicular syndrome [10].

### 3.2. HIV and Common Dermatoses

Some common dermatologic disorders such as seborrheic dermatitis are reported with a higher frequency in HIV patients than the general population. Some others, including atopic-like dermatitis, psoriasis, pityriasis rubra pilaris (PRP), Reiter's disease, and hidradenitis, have also been demonstrated to be related to AIDS (Figures 1(a)–1(e) and 2(a)–2(d)).

HIV-related seborrheic dermatitis can be more severe and resistant to usual therapies such as less potent topical corticosteroids and topical azoles, with more frequent relapses. Moreover, scales are usually thicker and more yellowish and erythroderma also occurs more commonly in HIV-related SD [11]. However, most cases of HIV-associated seborrheic dermatitis regress with ART; therefore, timely detection of HIV infection can improve the prognosis [12].

An atopic-like dermatitis is quite common in HIV patients, especially pediatrics. This condition is more common in those with familial history of atopy. It should be kept in mind that in any case of adult-onset or generalized eczematous skin with lymphadenopathy and constitutional symptoms, HIV infection should be excluded [13].

Psoriasis is among the relatively common dermatoses in HIV patients. Psoriasis may be more severe in HIV patients; nails involvement is more common and prominent in HIV-related psoriasis. Moreover, it may be pruritic and accompanied by arthritis in this population. Another distinguishing characteristic of HIV-associated psoriasis from the classic form is the increased probability of concurrence of multiple morphological types in a single patient. HIV-related immune dysregulation may be responsible for psoriasis onset in the settings of this infection [14].

Management of HIV-associated psoriasis is challenging but is mainly controlled with ART. Methotrexate has been used for psoriatic arthritis, but care should be taken in administering this agent in HIV patients due to the probability of inducing leukopenia and mortality increase, especially if coadministered with trimethoprim/sulfamethoxazole. Antitumor necrosis factor-*α* (anti-TNF-*α*) agents such as etanercept have been used effectively in psoriatic arthritis especially in HIV patients; nevertheless, increased risk of polymicrobial superinfections should be considered [15].

Pityriasis rubra pilaris (PRP) is a rare papulosquamous dermatosis, mostly presenting as a component of HIV-associated follicular syndrome, including hidradenitis suppurativa, acne conglobate, and lichen spinulosus. Aberrant immune response to HIV antigens and the resultant follicular inflammation is proposed to be the underlying mechanism of HIV-related PRP. It is more common in HIV-related PRP to present with disfiguring and atypical patterns such as horny perifollicular mucinosis, explosive cystic acne, lichen spinulosus, and nodulocystic lesions with occasional dystrophy of the nails. PRP may have poorer prognosis and present with fatal complications such as cutaneous sepsis in HIV patients. Moreover, the risk of squamous cell carcinoma (SCC) is increased in HIV-associated PRP [16].

Hidadenitis suppurativa (HS), also known as acne inversa can be triggered in the context of HIV infection. A certain type of HS, known as neutrophilic eccrine hidradenitis (NEH), is proposed to be associated with chemotherapy-induced neutropenia but has also been reported in the settings of HIV infection. It is manifested as erythematous plaques on unusual locations such as the face, trunk, or limbs [17].

### 3.3. Opportunistic Skin Infections

In advanced stages of HIV infection with lower CD4 counts and higher vial loads, most of the patients develop opportunistic infections including herpetic and mycobacterial infections, cryptococcosis, histoplasmosis, molluscum contagiosum, and bacillary angiomatosis (BA) (Figures 3(a)–3(g)). It is important to know that these opportunistic infections may present differently in the settings of AIDS.

Molluscum contagiosum is a viral cutaneous infection resulted from a poxvirus. This viral infection is per se a mild, self-limiting disease with skin umbilicated papules; however, a disseminated giant form, disfiguring one, or a form involving atypical locations such as the face and eyelids may be demonstrated in HIV patient‏s.

Histoplasmosis is a granulomatous infection caused by *Histoplasma capsulatum*. This dimorphic fungus mainly involves the lungs; however, cutaneous manifestations are common in HIV patients either as an isolated cutaneous histoplasmosis or as a component of disseminated histoplasmosis. Furthermore, it may present as a feature of IRIS.

*Talaromyces marneffei* (previously called *Penicillium marneffei*) is a systemic mycosis usually reported in immunosuppressed individuals such as AIDS patients. However, it is mostly observed in endemic areas such as China and Southeast Asia. This opportunistic infection most commonly presents with generalized molluscum contagiosum-like papular lesions, which is sometimes difficult to distinguish from cyptococcosis and histoplasmosis [18].

Sporotrichosis is another fungal infection that mainly presents with cutaneous lesions in immunocompetent individuals; however, disseminated cutaneous or systemic involvement is quite common in immunocompromised patients including HIV-infected individuals [19].

Cryptococcus skin involvement usually occurs in the settings of disseminated cryptococcosis. This condition should be suspected in any HIV-infected patient presenting with umbilicated papules, acneiform pustules, or papulonodular necrotizing skin lesions such as molluscum contagiosum [20].

Cutaneous tuberculosis (TB), caused by mycobacterium tuberculosis, has a wide range of presentations which include papulonecrotic tuberculid, tuberculous chancre, lupus vulgaris, tuberculosis verrucosa cutis, orificial tuberculosis, scrofuloderma, lichen scrofulosorum, erythema induratum of Bazin, nodular granulomatous phlebitis, miliary tuberculosis, and metastatic tuberculosis abscess (gummatous tuberculosis). Early diagnosis and management of cutaneous TB is particularly important in the settings of disseminated miliary tuberculosis, which has a poor prognosis [21].

Nontuberculous mycobacteria (NTM) which have been reported to induce cutaneous lesions in HIV patients include *Mycobacterium avium complex, M. kansasii, haemophilum, colombiense, shigaense, and malmoense*. Clinical features of cutaneous NTM include panniculitis, ecthyma gangrenosum-like lesion, recurrent cellulitis, erythematous papules, plaques or nodules, and Buruli ulcer [22].

Herpes zoster (HZ) virus infection (shingles) is defined as reactivation of latent *Varicella zoster virus (*VZV) infection in neuronal ganglions. HIV patients are generally at increased risk of herpes zoster infection with atypical presentations such as necrotic or verruciform hyperkeratotic lesions. It has been postulated that genital zoster may be an early indicator of HIV infection. However, its appearance in HIV-positive individuals is not a predictor of poorer prognosis but lesions with faster evolution, more severity, and prolonged duration are expected; the risk of complications such as PHN, ophthalmic involvement, superinfection, and CNS sequels is also increased in the settings of HIV, particularly advanced infection [23].

Bacillary angiomatosis (BA) is caused by *Bartonella quintata and B. henselae*.

*Bartonella henselae* tend to induce superficial skin lesions, while *B. quintana* is more likely to cause subcutaneous, deep, and lytic bone lesions.

Cutaneous leishmaniasis (CL), caused by the protozoa leishmania species, is not per se an opportunistic infection, but its reactivation or worsening can be more common in immunosuppressed individuals. Visceral leishmaniasis can also spread and cause skin lesions in the settings of HIV [24].

### 3.4. IRIS-Related Skin Manifestations

Despite the beneficial effects, the introduction of ART has brought about unfavorable consequences, among which we can name immune reconstitution inflammatory syndrome (IRIS), which is the result of an exaggerated inflammatory response against some opportunistic infections. This condition is caused by some infection flare-ups or paradoxical clinical worsening [25].

### 3.5. HIV and Ectoparasitic Infestation

Due to poor general hygiene and homelessness of many of HIV-infected individuals, this population is at an increased risk of ectoparasitic infestation with scabies and pediculosis [26].

Scabies can present atypically with a highly contagious and fulminant form known as “Norwegian scabies” or “crusted scabies,” which is characterized by diffused or localized thick, friable plaques and sometimes with diffuse erythoderma (Figure 3(b)). In addition, nodular scabies is also common in the settings of HIV infection. Crusted scabies is more common in advanced immunosuppression; however, it can occur in any stage of HIV infection.

The use of lindane is exceptionally discouraged in HIV patients with crusted scabies because of the increased risk of neurotoxicity. Norwegian scabies is usually resistant to conventional topical antiscabies therapies due to the hyperkeratosis in the crusts; therefore, crusts should be removed with the use of keratolytic agents such as lactic acid, urea or salicylic acid, and manual debridement [27].

### 3.6. STDs with Dermatologic Manifestations

The HIV epidemic has revealed many unknown aspects of sexually transmitted diseases (STDs). It is important to know that HIV infection is not only an STD per se but also can alter the clinical manifestations and management of other STDs, while other STDs, especially genital ulcers, can increase the risk of acquiring and transmission of HIV (Figures 4(a)–4(g)).

Ulcerative STDs include syphilis, chancroid, genital herpes, lymphogranuloma venereum, and granuloma inguinale, while nonulceative STDs include genital human papillomavirus infection (warts), molluscum contagiosum, secondary syphilis, and ectoparasitic infestations. Traumatic or violent sex and male homosexuality increase the risk of HIV transmission [28].

Every STD has its own specific characteristics; however, the clinical manifestations and course may be affected by HIV infection. This can be either modification or intensification of STD lesions. Clinical course and management of STDs may also be affected in the settings of HIV infection.

### 3.7. IVDU and Cutaneous Complications

Apart from sexual contact, HIV is also transmitted through injection drug abuse. Intravenous drug users (IVDUs) are at an increased risk of certain cutaneous infections that are unique in this population. Unsterile substances and equipment, poor hygiene, and special injecting practices such as intradermal injection or skin popping are risk factors of skin and soft tissue infections in IVDUs. Poly microbial skin infections are quite common in these individuals.

Cellulitis, abscesses, necrotizing fasciitis, granulomas, phlegmons, nonhealing ulcers, purpura, microemboli, and leukocytoclastic vasculitis occur commonly in IVDUs. Other dermatologic conditions that have been demonstrated in IVDUs include necrobiosis lipoidica-like dermatitis, dermal pigment deposition, nodules, panniculitis, sclerosis, scars, shooting tattoos, and acneiform skin lesions [29].

Wound botulism and tetanus are other possible infections in IVDUs which are caused by *Clostridium botulinum and C. tetani*, respectively [30].

Injectional anthrax, caused by *Bacillus anthracis*, is a type of anthrax most commonly occurring in IVDUs, particularly heroin skin poppers. This cutaneous infection is characterized with painless skin ulcers with a black center and marked peripheral edema. The significance of this type of anthrax is its potential to rapidly progress to septicemia and death [31].

### 3.8. Vasculitis

Vasculitis has been a rare phenomenon in the settings of HIV infection; however, this uncommon event, particularly those involving small and medium-sized vessels, can present with dermatologic manifestations. Among small vessel vasculitis, we can mention hypersensitivity vasculitis (sometimes associated with ART and other medications used in HIV-infected patients), erythema elevatum diutinum, and mixed cryoglobulinemia. Medium-sized vessel vasculitis with cutaneous manifestations includes polyarteritis nodosa and Kawasaki-like syndrome. On the other hand, some vasculitides are associated with known infectious agents in the settings of HIV infection; as such we can name hepatitis B virus (polyarteritis nodosa, serum sickness, Gianotti-Crosti syndrome, or papular acrodermatitis), hepatitis C virus (essential mixed cryoglobulinemia), and cytomegalovirus (erythematous maculopapular or purpuric eruptions) [32].

## 4. Metabolic Diseases

Metabolic abnormalities are also prevalent in HIV patients. The hallmark of these metabolic changes is depicted in the form of lipodystrophy. This syndrome is usually related to protease inhibitors (PIs) use as a component of ART. Symmetrical lipomatosis, breast hypertrophy, and dorsocervical fat deposition are other metabolic changes attributable to PIs [33].

### 4.1. AIDS-Related Skin Malignancies

Although the majority of HIV-related skin diseases are of infectious and inflammatory origin, skin cancers do occur in the settings of HIV infection, especially in advanced immunosuppression (Figures 5(a)–5(e)). These cutaneous malignancies include Kaposi sarcoma, cutaneous lymphomas such as B-cell non-Hodgkin lymphoma and cutaneous T-cell lymphoma, and melanoma.

Kaposi's sarcoma (KS) usually occurs in late stages of HIV infection, but it can happen in any CD4 count or HIV viral load. Kaposi sarcoma inflammatory cytokine syndrome (KICS) is a relatively new syndrome described in patients with HIV and KS which clinically resembles multicentric Castleman disease (MCD). In fact, both of these entities present with various degrees of lymphadenopathy, pancytopenia, HIV and KS, and signs of systemic inflammatory syndrome (SIRS). However, KICS has higher mortality. Lymph node, bone marrow, or splenic biopsy can help differentiate between the 2 entities [34].

### 4.2. ART-Associated Drug Eruptions

Although introduction of ART has led to decreased incidence of HIV-related cutaneous disorders, the antiretrovirals (ARVs) themselves have been the cause of several skin drug reactions, especially due to administration of multiple drugs simultaneously to a single patient [35].

In general, drug reactions that have been demonstrated with ART, range from mild exanthematous reactions to Stevens-Johnson syndrome (SJS)/toxic epidermal necrolysis (TEN). SJS mainly occurs in those HIV-infected patients who are receiving sulfa-drugs such as trimethoprim-sulfamethoxazole (Figures 6(a)–6(d)).

Nucleoside reverse transcriptase inhibitors (NRTIs) such as zidovudine, lamivudine, and stavudine are known to induce drug hypersensitivity (DRESS), mucocutaneous pigmentation, SJS, and leucocytoclastic vasculitis. Non-nucleoside reverse transcriptase inhibitors (NNRTIs) such as nevirapine and efavirenz are commonly reported to induce exanthematous eruption and drug-induced toxic epidermal necrolysis (Lyell syndrome). Interestingly, some cases of familial occurrence of SJS/TEN associated with nevirapine have been described, suggesting a genetic basis [36]. Although no HLA or genetic basis has been determined in many studies (245), it has been suggested that the phenotype of the drug hypersensitivity syndrome may have impact on presentations related to specific HLA associations. For instance, HLA − DRB1 ∗ 0101 was associated with rash only in the presence of hepatitis [37].

Protease inhibitors such as indinavir and ritonavir may cause pruritic maculopapular eruption, asteatotic dermatitis, lipodystrophy, pyogenic granuloma, acute porphyria, SJS, and DRESS [38].

In conclusion, cutaneous manifestations in HIV-infected patients are quite various, ranging from AIDS-specific skin eruptions, opportunistic infections, IRIS-related skin manifestations, exacerbation or altered pattern of common dermatosis, nonopportunistic skin infections, ectoparasitic infestation, other STDs with dermatologic manifestations, IVDU and cutaneous complications, ART-associated drug eruptions to AIDS-related malignancies, and vasculitis.

Most of the cutaneous disorders in the context of HIV/AIDS are managed easily in outpatient settings; however, some of them cause significant morbidity and mortality.

This study would be useful in understanding the various specific and nonspecific features of AIDS-associated cutaneous manifestations, helping the clinician to manage these conditions.

## 5. Transparency Declaration

Authors declare that the manuscript is honest, accurate, and transparent. No important aspect of the study is omitted. Patients and Public Partnership Patients or the public were not involved in the design, or conduct, or reporting, or dissemination plans of our research.

### 5.1. Permission to Reproduce Material from Other Sources

We used images of some patients visited by one of the authors (N.E.) in Africa (Figure 1). Written informed consents were obtained from the patients for publication of their clinical data and any accompanying images.

## Data Availability

The data that support the findings of this study are available from the corresponding authors upon reasonable request.
